# Scalp eschar and neck lymphadenopathy by *Rickettsia slovaca* after *Dermacentor marginatus* tick bite case report: multidisciplinary approach to a tick-borne disease

**DOI:** 10.1186/s12879-021-05807-3

**Published:** 2021-01-22

**Authors:** Giulia Barlozzari, Federico Romiti, Maurizio Zini, Adele Magliano, Claudio De Liberato, Franco Corrias, Guglielmo Capponi, Luisa Galli, Manuela Scarpulla, Carlotta Montagnani

**Affiliations:** 1 Istituto Zooprofilattico Sperimentale del Lazio e della Toscana M. Aleandri, Rome, Italy; 2 Istituto Zooprofilattico Sperimentale del Lazio e della Toscana M. Aleandri, Florence, Italy; 3 Post-Graduate School of Paediatrics, Department of Health Sciences, Anna Meyer Children’s University Hospital, Florence, Italy; 4 Department of Health Sciences, University of Florence, Florence, Italy; 5 Infectious Disease Unit, Anna Meyer Children’s University Hospital, Viale Pieraccini 24, IT-50139 Florence, Italy

**Keywords:** SENLAT, DEBONEL, *Rickettsia slovaca*, *Dermacentor marginatus*, Tick-borne rickettsioses, Case report

## Abstract

**Background:**

Scalp Eschar and Neck LymphAdenopathy after Tick bite is a zoonotic non-pathogen-specific disease most commonly due to *Rickettsia slovaca* and *Rickettsia raoultii.* Diagnosis is mostly based only on epidemiological and clinical findings, without serological or molecular corroboration.

We presented a clinical case in which diagnosis was supported by entomological identification and by *R. slovaca* DNA amplifications from the tick vector.

**Case presentation:**

A 6-year-old child presented with asthenia, scalp eschar and supraclavicular and lateral-cervical lymphadenopathy. Scalp Eschar and Neck LymphAdenopathy After Tick bite syndrome following a *Dermacentor marginatus* bite was diagnosed. Serological test on serum revealed an IgG titer of 1:1024 against spotted fever group rickettsiae, polymerase chain reaction assays on tick identified *Rickettsia slovaca*. Patient was successfully treated with doxycycline for 10 days.

**Conclusions:**

A multidisciplinary approach including epidemiological information, clinical evaluations, entomological identification and molecular investigations on tick, enabled proper diagnosis and therapy.

## Background

SENLAT (Scalp Eschar and Neck LymphAdenopathy after Tick bite) is a zoonotic non-pathogen-specific disease characterized by enlarged neck lymph nodes and scalp eschar after a tick bite. *Rickettsia slovaca* and *Rickettsia raoultii* are the etiological agents most commonly responsible for this syndrome. Nevertheless, *Coxiella*-like bacteria are increasingly identified in SENLAT patients, while other bacteria such as *Rickettsia rioja*, *Rickettsia sibirica mongolitimonae*, *Rickettsia massiliae*, *Bartonella henselae*, *Coxiella burnetii*, *Borrelia burgdorferi* and *Francisella tularensis* are isolated only sporadically [1]. Ticks of *Dermacentor* genus are most frequently implicated in the transmission and human cases, mainly involving children or women, usually occur during the cold season (autumn and spring), when this tick species is most active [1]. Hence, the syndrome is also known as DEBONEL (DE*rmacentor*-BOrne Necrosis Erythema and Lymphadenopathy).

The syndrome is one of the most common tick-borne rickettsiosis in Europe, ranking second only to Mediterranean spotted fever (MSF), that is caused by *Rickettsia conorii* [2]. Cases of tick-borne lymphadenopathy have previously been reported in Tuscany (Italy) and *R. slovaca*-positive ticks found in wild boars (*Sus scrofa*) were detected in four Italian regions (Liguria, Sardinia, Tuscany and Abruzzo), pinpointing the potential eco-epidemiological role of this species as wild reservoir host [3–5]. Unlike *R. conorii* infection and other rickettsioses, SENLAT is characterized by localized dermatological manifestations (erythema and eschar) and milder symptoms, even though severe manifestations have been described, especially in untreated patients [1, 3]. Scarring alopecia on the bite site and chronic asthenia, lasting up to several months, are the sequelae especially in case of *R. slovaca* infections [1, 6]. Diagnosis of SENLAT is mainly based on epidemiology and clinical features. Although epidemiological and clinical characteristics, as well as severity, differ between *R. conorii* and *R. slovaca*, the interpretation of serological data can be confounding, due to the wide cross-reactivity that occurs among spotted fever group (SFG) rickettsiae. Therefore, the proper identification of *Rickettsia* species through direct methods, such as polymerase chain reaction (PCR) or culture, is necessary for a proper diagnosis. However, these methods are not commonly accessible, their sensitivity on clinical samples is variable and ticks are rarely available for being tested [1].

Hence, we describe a case of SENLAT in which proper diagnosis was obtained merging entomological identification, molecular investigation on tick and clinical features.

## Case presentation

**Fig. 1:**
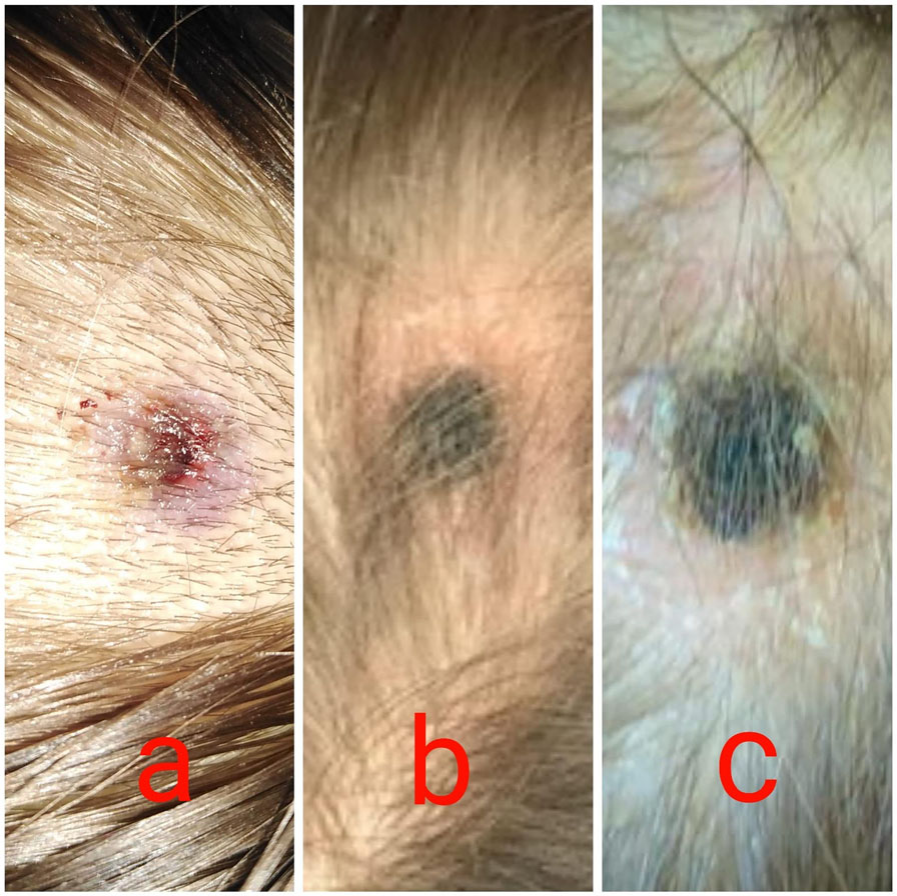
Evolution of the scalp lesion. **a** Fibrinous membrane and skin erythema surrounding the bite site of *D. marginatus* (10 days after tick bite). **b** Black eschar surrounded by a slight erythema (40 days after tick bite), **c** Resolution of erythema (2 months after tick bite)

In April 2020, a 6-year-old child presented with mild asthenia and nuchal pain not associated with fever (body temperature 37.2 °C). The mother found a large tick on the girl’s scalp, probably acquired walking in the countryside near Florence (Tuscany, Italy) 10 days earlier. The ectoparasite was successfully removed by the mother and sent to Istituto Zooprofilattico Sperimentale del Lazio e della Toscana M. Aleandri to be identified and tested for the presence of tick-borne pathogens. On physical examination by a pediatrician, a bite lesion on the scalp surrounded by skin erythema was revealed, while erythema marginatum was not evident (Fig. 1a). A 10 days antibiotic treatment with amoxicillin-clavulanic acid (25 mg/kg twice a day) was prescribed.

Tick was morphologically identified using taxonomic keys as an adult engorged female of *Dermacentor marginatus* [7]. An automated DNA extraction was performed by QIAcube platform using QIAamp cador Pathogen Mini Kit (Qiagen, Hilden, Germany).

PCR protocols targeting *glt*A, *omp*A and *omp*B genes were carried out to reveal the presence of SFG rickettsiae, as described previously [8]. PCR assays targeting *B. burgdorferi* sensu latu (s.l.) complex, *C. burnetii* and *Anaplasma phagocytophilum* were performed following current methods. PCR assays targeting *glt*A, *omp*A and *omp*B yielded positive results, while no amplification was obtained for other assays. The amplicons of expected size were then purified and sequenced bi-directionally according to BigDye 1.1 technology, using ABI3500 capillary sequencer (Applied Biosystems, Carlsbad, CA, USA). The resulting chromatograms were analyzed and edited using Geneious software (Biomatters Ltd.). The sequences obtained were compared to those previously deposited in GenBank by using the nBLAST algorithm (https://blast.ncbi.nlm.nih.gov/Blast.cgi). The amplified *glt*A, *omp*A, and *omp*B sequences were deposited in GenBank using BankIt (https://www.ncbi.nlm.nih.gov/WebSub/), acc. no. MT981150, MT981151, MT981152 (BankIt 2,363,297). These sequences showed an identity between 99.7 and 100% with GenBank acc. no. KJ663736 for *glt*A, HM161798 for *omp*A and KJ663756 for *omp*B of *R. slovaca*, isolated from ticks removed from human patients or free-living ticks in Italy.

Following the detection of *R. slovaca* in the tick, 40 days after the tick bite, the child was evaluated at the Infectious Disease Unit of Meyer Children’s University Hospital, Florence, Italy. Blood samples were collected for a complete blood count, serum chemistry, serological test for SFG rickettsiae and other common tick-borne pathogens. The scalp lesion had meanwhile evolved into a black eschar surrounded by a slight erythema (Fig. 1b), while left supraclavicular and left lateral-cervical lymphadenopathy was evidenced. White blood cell count was within the reference range and no rising of C reactive protein was detected. An indirect chemiluminescence immunoassay (CLIA) (LIAISON® systems, DiaSorin, Vercelli, Italy) was carried out to detect class M and class G immunoglobulins (IgM and IgG) against *B. burgdorferi* s.l. complex. The presence of IgG antibodies against R*. conorii* was checked using a commercial immunofluorescent-antibody test (IFAT) (Daltec Instrument s.r.l, Milano, Italy). CLIA for *B. burgdorferi* s.l. was negative, while an IgG antibody titer of 1:1024 against *R. conorii* was detected by IFAT. Western blot was not available.

Considering clinical features, tick identification, molecular and serological results, diagnosis of SENLAT was performed.

After obtaining written informed consent from the mother, a targeted off-label antibiotic treatment with doxycycline (2.2 mg/kg twice a day) for 10 days was prescribed. No side effects were reported during the therapy. At 2-week follow up, 2 months after the tick bite, erythema surrounding the scalp lesion disappeared with persistence of black eschar (Fig. 1c) and all neck lymph nodes were reduced in size on palpation. Asthenia gradually improved.

## Discussion and conclusions

Bite site (child’s scalp), season and location of the encounter with the questing tick (early April/countryside near Florence) and duration of feeding activity (10 days) are consistent with *D. marginatus* ecology. Indeed, this species is widely distributed in prairies and steppes of central Italy up to 2500 m above sea level. The adults are active from autumn to spring, parasitizing wild or domestic ungulates and they can occasionally feed on humans [5, 7]. *D. marginatus* can feed for several days, increasing the risk of pathogen transmission, and it is considered the main vector of SENLAT in our country [6]. The tendency of *Dermacentor* spp. to bite children or women on the scalp can be explained by the host preference for hairy animals and the questing height of 1–1.5 m on vegetation, typical of this genus [9]. Unfortunately, as described in this report, the diagnosis of SENLAT can often be delayed, because the bite lesion is usually hidden by hair and the symptoms are usually non-specific [2, 6, 10]. In our patient, the tick had been feeding for 10 days before being removed from the child’s scalp, thus having enough time to transmit the pathogen. Moreover, during this time no symptoms were evidenced, since the incubation period of the syndrome ranges from 1 to 55 days after the tick bite (typically between 5 and 10 days) [1]. Although the suspected diagnosis of tick-borne rickettsioses is based on epidemiological and clinical findings, serological tests are useful to confirm the exposure to a specific pathogen, as different agents (i.e., *B. burgdorferi* s.l., *F. tularensis*, *B. hensalae*, *C. burnetii*) could sometimes emerge with the same symptoms [6]. However, an antibiotic therapy needs to be started while waiting for laboratory confirmation, in order to prevent severe forms [5, 6]. IFAT is the reference serological method for SFG rickettsiae, but it could lack diagnostic sensitivity in the case of SENLAT, probably due to the local diffusion of bacteria. Thus, the identification of the specific agent on clinical samples (skin swabs or biopsies, crusts, blood or sera) or ticks by PCR or culture is required [1]. In this report an increase in IgG antibody titer (1:1024) against *R. conorii* was revealed by IFAT, confirming the low specificity of this technique, due to the frequent cross-reactions among SFG rickettsiae. In case of positive IFAT results a Western blot with cross-adsorption should be performed to discriminate the species involved [1]. The diagnosis of MSF was unlikely in our patient, as this syndrome is typically associated with other specific symptoms like acute fever, chills, headache, photophobia, arthralgia, muscular pain and maculopapular rash on palms and soles or less frequently on trunk. Moreover, *R. slovaca* DNA was successfully amplified and sequenced from the tick vector.

Treatment with beta-lactams is not effective against tick-borne rickettsiosis. Hence, in case of suspicion of tick-borne diseases, doxycycline should be preferred. Use of tetracycline in pediatric patients has historically been limited because of risk of permanent tooth discoloration in children younger than 8 years of age. However, doxycycline binds less readily to calcium compared with other tetracyclines and recent data showed that short treatment courses of doxycycline (less than 21 days) are not likely to cause teeth abnormalities [11]. For that reasons, American Academy of Pediatrics now recommends use of doxycycline regardless of patient age [12].

This case emphasizes the importance of collaboration between experts who, through a multidisciplinary approach including epidemiological information, clinical evaluations, entomological identification and molecular investigations on tick, can allow for a proper diagnosis and therapy of SENLAT and other tick-borne diseases. In absence of the tests performed on tick, diagnosis would not have been confirmed, since Western blot for discriminating SFG rickettsiae is not available in our centre.

Hence, in case of tick bite, patients should be advised to preserve the vector for further analysis in the event of developing symptoms and collaboration between different experts should be implemented.

## Data Availability

The amplified *glt*A, *omp*A, and *omp*B sequences were deposited in GenBank using BankIt (https://www.ncbi.nlm.nih.gov/WebSub/), acc. no. MT981150, MT981151, MT981152 (BankIt 2363297).

## Abbreviations

CLIA: Chemiluminescence immunoassay
DEBONEL: DE*rmacentor*-BOrne Necrosis Erythema and Lymphadenopathy
IFAT: Immunofluorescent-antibody test
MSF: Mediterranean spotted fever
PCR: Polymerase chain reaction
SENLAT: Scalp Eschar and Neck LymphAdenopathy after Tick bite
SFG: Spotted fever group
